# Autochthonous Human Schistosomiasis, Malaysia

**DOI:** 10.3201/eid1908.121710

**Published:** 2013-08

**Authors:** Baha Latif, Chong Chin Heo, Rahimi Razuin, Devi V. Shamalaa, Dennis Tappe

**Affiliations:** Universiti Teknologi MARA, Shah Alam, Malaysia (B. Latif, C.C. Heo);; Hospital Sungai Buloh, Sungai Buloh, Malaysia (R. Razuin, D.V. Shamalaa);; University of Würzburg, Wuerzburg, Germany (D. Tappe)

**Keywords:** Schistosoma malayensis, schistosomiasis, Malaysia, zoonoses, public health, ecotourism, parasite

**To the Editor:** In Malaysia, the only histologically diagnosed autochthonous cases of human schistosomiasis were reported in the 1970s, all in rural aborigine (Orang Asli) populations (*1*–*3*) (Technical Appendix Figure 1). The fact that the infection had been found only among aborigines had led to the proposal of a distinct unknown schistosome with an animal reservoir causing sylvatic infections (*2*,*3*). Consequently, during the 1980s, *Schistosoma malayensis* n. sp. was described from intermediate snail (*Robertsiella* sp.) and final mammalian hosts (*Rattus muelleri* and *R. tiomanicus* [*4*]). *S. malayensis* is closely related to *S. mekongi* and differs genetically from the latter by ≈10%. Both species differ from *S. japonicum* by 25% (*5*), and adult and ova morphologies are similar (*4*). Few transmission sites for this new *S. japonicum*–complex schistosome species were identified in rural areas (*4*). We report after 30 years the histologic finding of *S. malayensis*–like eggs in the liver of a Malay man and discuss public health implications.

A 29-year-old male nonaboriginal Malay from Subang Jaya in Selangor State, Peninsular Malaysia, had died suddenly of an intoxication in 2011. According to his mother, he had reported hematuria and dysuria during adolescence. Similar symptoms had reoccurred 10 years later, accompanied by constipation. The patient had never been outside of Malaysia, and he had gone bomb fishing for many years in Sungai Lepar Utara, a river near his village (Felda Tekam Utara, Jerantut, Pahang; 3°52’30”N, 102°49’2”E). No tests on blood or feces were performed before his death. An autopsy was conducted in Sungai Buloh Hospital, and gross pathology showed a normal heart, kidneys, and brain. The lungs were edematous and congested. The liver also was congested, but no macroscopic lesions were seen. Toxicology investigations showed methadone and a derivative in his blood and urine. During a routine histologic examination, several granulomas with intensive lymphocyte, monocyte, and eosinophil infiltration surrounding clusters of ovoidal eggs were found in the liver (Figure; Technical Appendix Figure 2). Serial sectioning showed that the eggs contained miracidia and had the overall appearance of *S. malayensis*–like ova 50 μm long × 28 μm wide. The ova were not operculated and had no bipolar plugs; the thin yellowish shell was not striated, but a knob-like structure was seen laterally. Morphologic differential diagnoses included eggs of *Capillaria hepatica* (bipolar striated ova in liver), *Dicrocoelium* (slightly smaller operculated ova typically found in feces or bile), and the similar *Eurytrema* (thick-walled operculated ova in feces).

**Figure:**
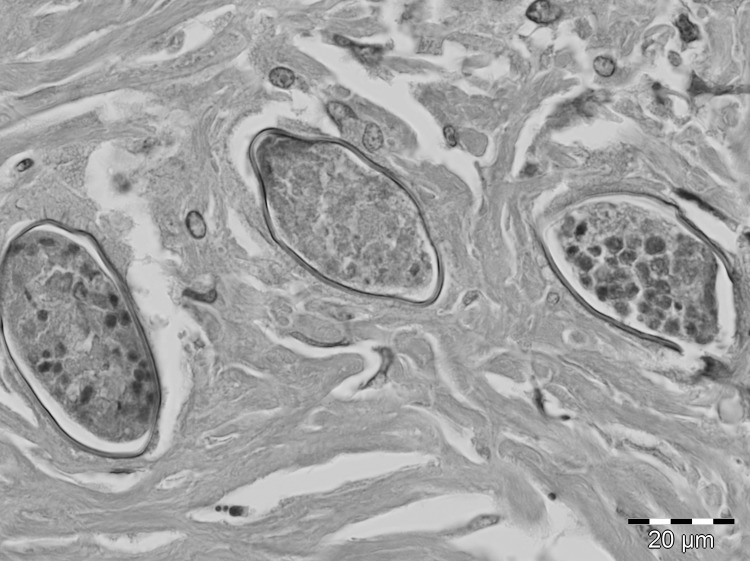
Close-up of liver granuloma with section through 3 *Schistosoma malayensis*-–like ova embedded in dense fibrous tissue. The thin-walled, nonstriated helminth ova are not operculated and contain nonvital miracidial cells. Hematoxylin and eosin stain; original magnification ×100.

Schistosomiasis is endemic in many developing countries and infects >207 million persons living in rural agricultural areas (*6*). In Asia, *S. japonicum*, *S. mekongi*, and *S. malayensis* cause human infection (*7*), with *S. japonicum* being the most dangerous. In Malaysia, *S. malayensis*, in addition to *S. spindale*, *S. nasale*, *S. incognitum*, *Trichobilhazia brevis*, and *Pseudobilharziella lonchurae*, is known to occur in wildlife (*8*). The first known case of human schistosomiasis in Malaysia was discovered in 1973 during an autopsy of an aborigine. *Schistosoma* eggs resembling those of *S. japonicum* were found in liver tissue (*1*). A subsequent retrospective autopsy study revealed additional cases with these *Schistosoma japonicum*–like ova in the rural aboriginal population, resulting in an overall prevalence of 3.9% (*2*). Several attempts to recover eggs from feces from the Orang Asli population in peninsular Malaysia (*3*), a biopsy-positive Orang Asli (*3*), and serologically positive persons (*9*, and others) were unsuccessful, however, which was attributed to the zoonotic nature of *S. malayensis* and thus missing adaptation to the human host. Whether hematuria, a typical sign of *S. haematobium* infection, as seen in the patient reported here also was caused by *S. malayensis* disease remains unclear because symptoms of the latter have not been reported. Serologic surveys for schistosomiasis in peninsular Malaysia showed prevalences of 4%–25% in selected rural populations (*9*). Because infected *Robertsiella* snails had been found almost exclusively in small rivers (*4*,*9*)—habitats like the Sungai Lepar Utara River in our current report—we suspect that the patient most likely became infected while fishing. The travel history may not be accurate because it was obtained from a relative, and possible unreported drug-related travel by the patient to neighboring countries cannot be fully excluded. *R. muelleri*, the jungle rat and definitive host for *S. malayensis*, is often seen at river banks (*4*), and rodent feces could have contaminated the water with schistosome eggs.

Future field studies are needed to identify focal hot spots of sylvatic transmission by snail examination and seroprevalence studies of persons living in rural areas, especially the Orang Asli population. Moreover, in light of growing ecotourism, which also encompasses stays at remote Orang Asli villages and canoeing on small streams (*10*), appropriate public health measures, such as rodent and snail control near tourist sites, should be implemented.

Technical Appendix Places of residence in Peninsular Malaysia of the schistosomiasis-infected Orang Asli from the 1970s and section of liver showing the helminth egg granuloma.
