# Supplementary material for: Autochthonous Human Schistosomiasis, Malaysia
Source: Emerg Infect Dis. 2013 Aug;19(8):1340–1. doi: 10.3201/eid1908.121710 (PMC3739520; doi:10.3201/eid1908.121710)
Supplement: Technical Appendix — Places of residence in Peninsular Malaysia of the schistosomiasis-infected Orang Asli from the 1970s and section of liver showing the helminth egg granuloma. [file 12-1710-Techapp-s1.pdf]

Article DOI: http://dx.doi.org/10.3201/eid1908.121710

Autochthonous Human Schistosomiasis, Malaysia

Technical Appendix

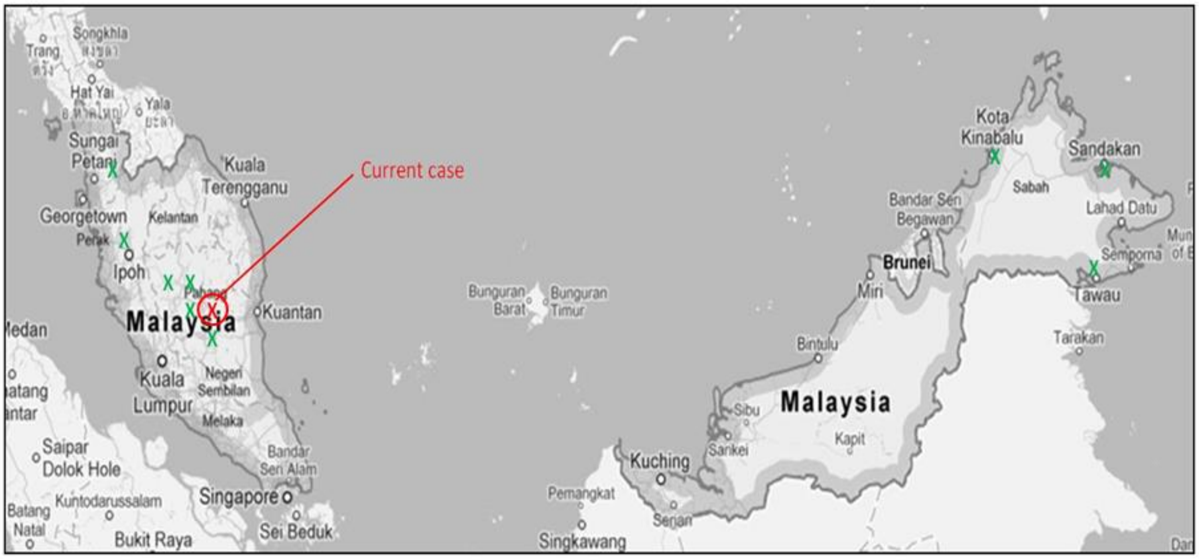

Technical Appendix Figure 1. Places of residence in Peninsular Malaysia (left) of the schistosomiasis-infected Orang Asli from the 1970s. The origin of the patient from the current report is marked with a red X in a circle; previous cases are marked with green Xs without circles. Three cases in persons also infected with *Schistosoma japonicum*–like eggs in Sabah, Malaysian Borneo (right), were immigrants and thus not considered autochthonous.

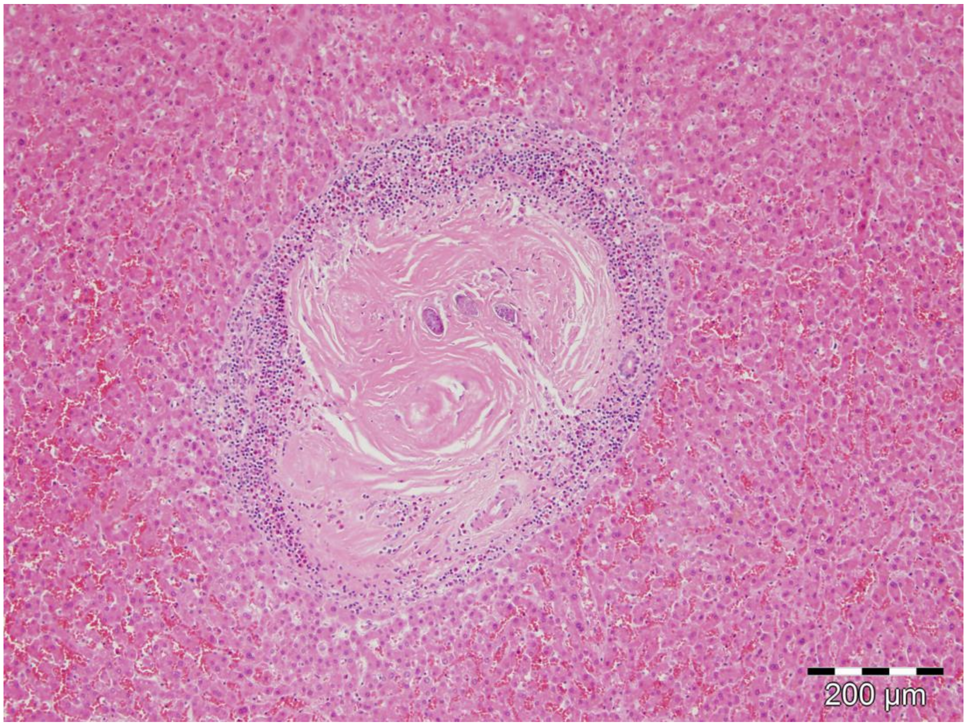

Technical Appendix Figure 2. Section of liver showing a helminth egg granuloma. The ova are embedded in fibrous tissue surrounded by a wall of eosinophils, lymphocytes, and monocytes. Hematoxylin and eosin stain, original magnification ×10.
